# Effects of Using Laser Technology for Cutting Polymer Films

**DOI:** 10.3390/ma17153678

**Published:** 2024-07-25

**Authors:** Małgorzata Olender-Skóra, Wacław Banaś, Marian Turek, Paweł Skóra, Aleksander Gwiazda, Krzysztof Foit, Agnieszka Sękala, Michał Stawowiak

**Affiliations:** 1 Department of Engineering Processes Automation and Integrated Manufacturing Systems, Faculty of Mechanical Engineering, Silesian University of Technology, Konarskiego 18A, 44-100 Gliwice, Poland; waclaw.banas@polsl.pl (W.B.); krzysztof.foit@polsl.pl (K.F.); agnieszka.sekala@polsl.pl (A.S.); 2 Department of Inorganic, Analytical Chemistry and Electrochemistry, Faculty of Chemistry, Silesian University of Technology, Krzywoustego 6, 44-100 Gliwice, Poland; marian.turek@polsl.pl (M.T.); pawel.skora@polsl.pl (P.S.); 3 Department of Mining Mechanization and Robotization, Faculty of Mining, Safety Engineering and Industrial Automation, Silesian University of Technology, Akademicka 2, 44-100 Gliwice, Poland; michal.stawowiak@polsl.pl

**Keywords:** laser cutting, manufacturing, automatization, robotization

## Abstract

In connection with the need to obtain a properly made and cut material and the appearance of the surface layer, new manufacturing technologies were used for tests, namely the laser cutting technology. This article describes the laboratory stand built for the purpose of research, as well as the possibility of using laser cutting on several sample materials (polymer films), together with an indication of the results obtained. The idea was to elaborate on the cutting technology that will be proper for manufacturing the desired type of spacers for ion-exchange membranes separating while maintaining the required level of product quality and chemical purity. The latter criterion was the basic one, due to the scope of use of the manufactured elements. This article also describes the problem encountered during the construction of the stand or during the research. The last part of this article describes the further steps of the research that will be carried out in the future along with a discussion and summary of the research performed. It is important from the point of view of the development of production technology, but also because of the characteristics of materials for the production of surface layers and coatings resistant to mechanical or thermal wear used in industry. The introduction of innovative solutions is also aimed at studying the improvement of the economics of the production of materials that are significant, in particular, for small- and medium-sized enterprises.

## 1. Introduction

In recent years, technology has changed, and this is also visible in the areas of production and management in various industries [1,2,3]. This is especially since an even faster execution of production orders is required, while maintaining the best possible production quality. The implementation of the production processes of appropriate elements also requires the selection of appropriate parameters of machines and devices, as well as time, which define, among other things, the production capabilities of enterprises. This is even more important if one considers the need for customization, which is currently very visible on the market. Customization is the production of a product in accordance with the requirements and preferences of an individual customer [4]. And it is precisely due to customer requirements, but also in effect available new manufacturing solutions, that manufacturers have more and more opportunities for different ways of business conducting. In addition to popular manufacturing methods, e.g., 3D printing [5,6,7,8], welding, and surfacing [9,10,11], laser cutting is also popular. This solution is useful because this method of processing can allow for cutting many materials, not only metals. This method uses the phenomenon of the thermal influence of laser light on materials [12]. Nowadays, lasers are widely used in many areas of life. Laser light is used during dental treatment, surgery, and esthetic medicine. In IT, a laser is an element that produces a stream of light that serves as an information carrier (CD/DVD/Blue Ray disks, fiber optic connections, etc.) or as a tool (optical scanners in mice, in the printing process in laser printers, etc.). In automation and metrology, laser light is used in measurement and control systems, in particular, to measure distances and perform three-dimensional scans of objects and the environment. In broadly understood mechanical engineering, lasers are used mainly in material processing technology (cutting, surfacing, drilling, engraving, etc.) and in reverse engineering (scanning machine parts).

This article focuses on one of the innovative manufacturing methods—laser cutting. The research problem was to design a laser head that would allow cutting delicate plastic materials (polyvinyl chloride films) while maintaining dimensional parameters and without burning the edges. Secondly, the goal was to achieve a certain level of flexibility of the cutting station, so that the shape of cut elements could be modified regardless of the material used (modulation of the laser beam power and control of the distance and speed of the head). In response to these requirements, especially regarding the cutting path, the laser was mounted on the robot arm. This solution provides greater possibilities of cutting and profiling the required shape in individual materials, and therefore specific applications of cut elements in specific chemical processes, i.e., the filtration of ionic liquids. Elements obtained by laser cutting are used in various types of processes, and therefore the surfaces and their edges must be damaged as little as possible (to avoid burns). Moreover, robotic laser cutting replaces the processes of manual cutting of materials, which were long-lasting, less effective, and unique. Mounting the laser on the robot arm allows you to automate the cutting process, thanks to which you can obtain a larger number of evenly cut elements in a shorter time. This also allows for increasing the economics of such a solution by using available infrastructure that is already implemented, especially by small- and medium-sized enterprises (having small industrial robots). Thanks to this, these companies are not forced to purchase new machines, and by using this solution, they can also become more competitive. This solution also allows for the personalization of products, as the built station and the ability to cut various shapes by programming cutting trajectories allow for high production flexibility.

This article also includes a short literature review on innovative manufacturing methods, e.g., cutting, which, in addition to methods such as welding or surfacing, is widely used.

The stand presented in this paper was entirely designed and constructed at the Department of Automation of Technological Processes and Integrated Manufacturing Systems, Faculty of Mechanical Engineering, Silesian University of Technology, Gliwice, Poland. The station consists of a robot (the department’s equipment); a laser (the department’s equipment), mounted to the robot using an adapter printed on a 3D printer [13,14,15]; and a laser power regulator controlling its power (also entirely made in the department) [16,17,18]. In addition, to show the possibility of cutting shapes of the stand, the appropriate shape was programmed in the controller. The combination of these solutions allowed for obtaining elements in which the surface of the material is important and should be damaged as little as possible, thanks to which these materials can be used in various processes where mechanical or thermal strength is important, such as in chemical processes.

The effect of laser cutting of materials currently used in filtering systems is also de-scribed. This is because the indicated station was used, among other things, to cut specialized, individual filter inserts used in ionic processes.

## 2. Review of Laser Cutting Technologies

Cutting materials referred to in article [19] are widely used in various industries. According to the authors, the emission of light using a laser is a complex phenomenon. A well-known, researched, and described problem in physics is the dual, particle-wave nature of light. Light is made up of particles called photons, and the motion of photons is a wave motion. A laser, on the other hand, is an artificial light source, just like a tungsten bulb, fluorescent lamp, or LED. An important element that distinguishes laser light from the other light sources mentioned above is its high light intensity, the order at the level of photons, and the directed nature of their movement. Thanks to the phenomenon described above, the laser light beam can be precisely directed at a given object or point in the object.

Sharma and Yadava [7] believe that the laser cutting process is carried out using various types of lasers, the most common of which are CO_2_ and Nd-YAG. The authors compared both types of lasers analyzed. In the research described in their paper, they showed that the Nd-YAG laser operates at a wavelength about ten times smaller than the CO_2_ laser, resulting in a shorter degree of beam reflection from metal surfaces. The Nd-YAG laser is also identified by more favorable cutting parameters in the pulse mode, but its undeniable advantages are smaller dimensions (compared to the CO_2_ laser) and the ability to transmit the beam via optical fibers, which is particularly important in mechanical and robotic applications.

In paper [1], Chryssolouris and Sheng, referring to the costs of the laser cutting process, stated that they must be balanced not only by achieving high dimensional accuracy and cutting quality but also by correspondingly high speed and repeatability.

On the other hand, Di Pietro and Yao in article [2] defined the following quality criteria for the laser cutting process: the width of the cutting gap, perpendicularity of the cutting edge, inclination of the inner surface of the crack, width of the heat-affected zone, appearance of the slag, and surface roughness, defined as the geometry of the resulting fringes (depth and distance between peaks). According to the authors, these elements can be used depending on the current needs. 

In article [3], Khosaim et al. presented a study of the influence of process parameters on the cutting gap characteristics during PMMA machining. 

In article [4], Oh et al. focused on evaluating the laser cut quality of CFRP (carbon fiber-reinforced plastic), analyzing the following indicators such as gap width and depth, die recession, and evaporation width, as well as damage zone, slot edge angle, and cut surface morphology. The authors related the observations to parameters such as laser power, scanning speed, and number of passes. In addition, Oh and others also described conclusions that determine the optimal conditions for the process. Article [5] presents research conducted by Choudhury and Shirley, where the authors used PMMA, PP, and PC materials. In turn, Eltawahni et al. in article [6] focused on the selection of a combination of parameters of the laser cutting process of MDF boards to obtain the highest quality of the cutting gap. In article [8], Siebert and others presented research on cutting sheet metal used to construct electrically powered machines. The authors pointed out an important difference between mechanical and laser sheet metal cutting, concerning the reduction in the magnetic flux and its distribution, depending on the technology adopted.

The laser cutting process is increasingly being robotized. For this purpose, special designs of manipulators can be used, as demonstrated in the literature [10,11], or typical industrial robots, which in turn was presented by the authors of articles [9,12], as is also the case with the research conducted in the department. This approach is characterized by high flexibility in terms of the path of movement along which the effector emitting the laser beam moves. This allows for making spatial cuts along surfaces of various shapes. However, issues related to the possible installation of the laser on the robot’s wrist should be considered, about the maximum payload of the manipulator, cable routing, and predicting the possibility of the collision of the effector with individual links of the kinematic chain.

Therefore, the problem, which also arose during the research, is related to the possibility of using modern technologies, i.e., laser cutting mounted on a robotic arm, but also to the minimization of economic aspects of production, due to, among other things, a greater possibility of using the available infrastructure, but also the saving of the cut material by reducing waste after cutting. In this regard, laser cutting is the best solution. This is due to the ability to use the laser anywhere in the company, without having to take up a huge space, as in the case of a plotter. Another advantage of this type of solution is the possibility of using different laser powers, which increases the flexibility of cutting, as well as the chances of obtaining custom production, which in turn was pointed out in article [20]. This is important because of the changing needs of customers, but also the opportunity for small- and medium-sized enterprises (SMEs) to grow. Another important issue is the ability to cut different types of materials, thus often replacing manual cutting, which is not always advisable, especially if they are complex shapes that must be within tolerances. Therefore, the advantage of laser cutting is the ability to cut complex shapes in the material, but also the further development of such a station.

The authors of article [7] point out the following problems related to the phenomenon that cutting sheet material is considered an important process, which is important among technical objects such as airplanes, ships, cars, furniture, etc. Based on recent research work in sheet metal cutting, it has been found that the Nd-YAG laser is used for sheet material processing. Article [7] reviews the experimental analysis of the Nd-YAG laser cutting process, carried out to determine the effect of laser cutting parameters on the efficiency index of the analyzed process. 

In article [21], the authors stated that laser cutting is one of the most widely used types of non-contact processing based on the process of generating thermal energy for cutting engineering materials such as titanium, stainless steel, aluminum, and aluminum alloys, as well as non-metallic materials in the form of wood, glass, plastics, ceramics, and composites used in various manufacturing industries. 

The content of article [22] refers to laser cutting of a variety of materials, including Ti-6Al-4V alloy, 304 steel, Inconel 625, and alumina, to evaluate changes in kerf width along the cut section. According to the authors, a life cycle assessment is carried out to determine the environmental impact of laser processing in terms of material waste during the cutting process. The kerf width size is formulated and predicted by an experimental analysis of solid parameters and measured by experiments. The effect of laser output power and laser cutting feed rate on kerf width variability is analyzed using analytical and optical tools, including scanning electron microscopes. In the experiments, the authors used a high-pressure nitrogen booster gas to prevent oxidation reactions in the cutting section. They found that the slit width size predicted by the analysis of the solid parameters agreed well with the experimental data. It is worth noting that the variation in kerf width increases with the increase in laser output power.

Article [23] describes a comprehensive review of research on the effect of laser cutting parameters on the quality of surfaces and metal kerfs.

Plastics such as titanium alloys, steel alloys, and aluminum alloys are significant materials due to their importance among everyday products as well as high-strength applications in aircraft, ships, automobiles, construction, military, and marine purposes. In recent times, laser cutting is one of the best and fastest unconventional methods of cutting sheet metal, so it is necessary to understand how laser cutting parameters affect the quality of the cut. A comprehensive overview is provided to investigate how laser cutting parameters affect the quality of the cut surface and kerf, and which parameters have the greatest impact on cut quality. An overview of the advantages of laser cutting compared to other machining methods is presented. In addition, a description of the laser cutting method and the different laser sources is presented, along with an explanation of the thickness range of the cut material for each source and their advantages. A description of the properties and applications of the materials studied is also discussed. The cutting performance parameters are illustrated in detail with graphs and equations. The analysis and research discussion are discussed in detail using tables and charts that show a complete classification of the studied works. It has been found that the best conditions to achieve low surface roughness, small HAZ width, small kerf width, and small kerf angle are to use low laser power, high cutting speed, medium gas pressure, long distance, medium pulse frequency, medium pulse width, small nozzle diameter, small thickness, and nitrogen as the auxiliary gas.

The authors of paper [24] presented a study in which a pulsed laser with a wavelength of 355 μm was used to cut silicon carbide wafers with a thickness of 355 μm. The morphology of the surface and changes in the elemental composition and structure of the cutting line after machining were analyzed by the authors using lasers of different power, pulse repetition frequency, scanning speed, and scan repetition settings. These results were then compared with those observed for a test sample made with a diamond blade. As a result of the analysis, it turned out that the average laser power oscillated around 6 W, the pulse repetition rate was 60 kHz, and the scanning speed of 5 mm/s minimized oxidation, which allowed for a groove width of 15.1–18.6 μm, narrower than the groove cut with a diamond blade. The authors noted that X-ray photoelectron spectroscopy showed that the heat-affected zone and oxygen content in the surface casting layer increased as the laser energy and number of repeats also increased as the scanning speed decreased. It is also worth noting that the oxygen content increased from 15.93% (diamond cutting) to 47.81%.

In article [25], the authors presented a method of optimizing laser cutting parameters for PMMA using metaheuristic algorithms. The study was based on the determination of optimal laser input parameters that simultaneously meet the required kerf width and depth during CO_2_ laser treatment of various polymethyl methacrylate (PMMA) sheets. The study of the analyzed sample was divided into three stages. The first is to model the PMMA cutting process by optimizing the polynomial curve as a function of laser power, laser speed, and distance. To measure the performance of the proposed model, the R-squared (R^2^), corrected R^2^, and mean squared error (RMSE) criteria were considered. The effect of laser parameters on the process was investigated by an analysis of variance (ANOVA) and sensitivity analysis. The second stage of the study is the optimization of derivative nonlinear regression models using the genetic algorithm (GA), particle swarm optimization (PSO), optimization algorithm (WOA), and optimization (ALO) and comparison of the performance of these algorithms. The third and final stage is to compare the adequacy of the optimization process with an artificial neural network (ANN). The research conducted by the authors has shown that the best-fitting polynomials are obtained with R^2^ and corrected R^2^ values of over 99% and 97%, respectively. The ANOVA algorithm and sensitivity test showed that the laser power sensitivity, which is the most effective parameter, was 150 W, at low powers, and decreased to 0 as the power value increased. When the nozzle spacing is 4.1, the proposed metaheuristics gave the authors effective and sufficiently accurate results. PSO stood out both in terms of the best cost value (3.49 × 10^−8^) and the relative value of measurement error of the order (0.19%). The authors found that the relative error of the ANN is 3% in terms of gap depth.

Taking into account all these advantages supporting the use of a laser for cutting, a research stand was built in the Department of Automation of Technological Processes and Integrated Manufacturing Systems of the Faculty of Mechanical Engineering of the Silesian University of Technology, Gliwice, Poland.

## 3. Description of the Station and Material to Be Cut

**Figure 1:**
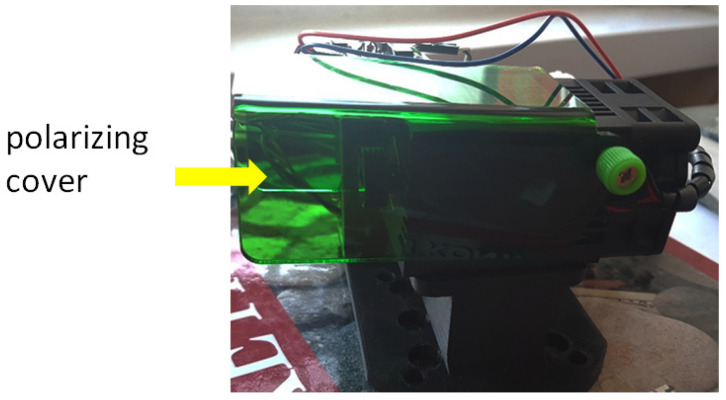
A view of the laser with the cover installed.

The laser cutting station consists of a Fanuc Arc Mate 100iB robot (equipped by the Department of Automation of Technological Processes and Integrated Manufacturing Systems, Silesian University of Technology, Gliwice, Poland), and a blue light-emitting laser (also equipped by the department). In the initial stages of testing, the laser was mounted without a special cover to protect users from the laser light, hence the need to wear special glasses. However, in later tests, the laser was equipped with a special protective cover, which from the users’ point of view is more functional during operation. However, the additional cover also had to be taken into account when programming the distance of the laser from the table. A diode laser was used to build the laser head. It is a blue light laser with a power of 20 W. The wavelength of blue light is 450 nm. The focal length of this laser is 20–40 mm (it is a variable focal laser). The thickness of the cut materials is on average 10–15 mm for plastics (depending on the hardness of the material). Larger cutting thicknesses require the use of air blowing. The diameter of the spot at the focal length is 0.1 mm. The view of the used laser with a shield is shown in Figure 1.

**Figure 2:**
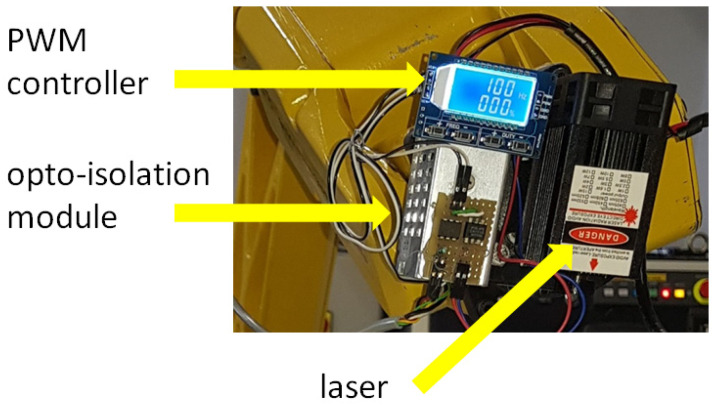
A view of the regulator made and mounted on the robot arm.

In turn, the laser was mounted to the robot arm using special adapters. These adapters were printed on a 3D printer [26,27]. Then, a laser power regulator was mounted on the arm to control its power (Figure 2). The regulator was also made in the department.

**Figure 3:**
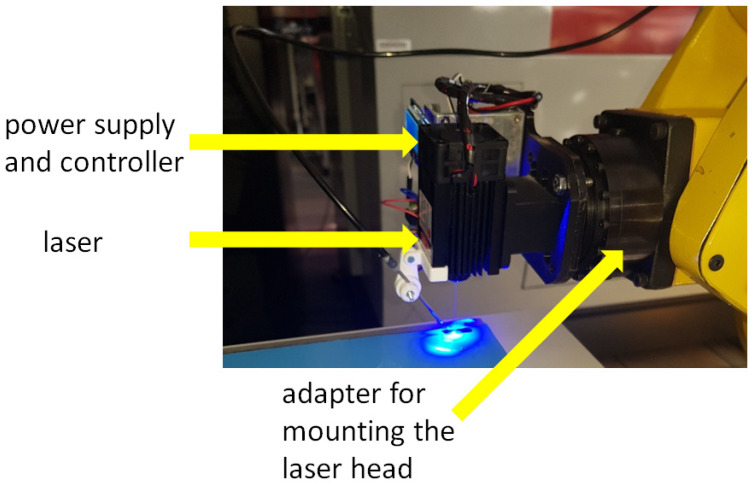
A view of the laser mounted on the robot arm.

The view of the laser mounted on the robot arm is shown in Figure 3.

Additionally, to eliminate one of the most important problems noticed during the tests, related to burning the edges of the material, an air supply was also installed at the station, thanks to which air was constantly supplied to the place of contact between the laser and the material using pneumatics (the mounting is also visible in Figure 3).

The stand prepared in this way at the department made it possible to carry out tests and finally cut out the ordered shape on the material provided by the Department of Inorganic, Analytical Chemistry and Electrochemistry, Faculty of Chemistry, Silesian University of Technology, Gliwice, Poland. The implementation of the cut, its effects, problems during testing, and the final result are described in the following sections.

## 4. Implementation of the Cutting Process

Laser cutting is used in many areas. Depending on the laser power, various types of materials can be cut. Due to the adaptability of available solutions, this technology can also support small- and medium-sized enterprises due to the customization of products, whether made of composites or metals.

Due to the need to obtain a properly made and cut material and the appearance of the surface layer, new manufacturing technologies were used for research, namely laser cutting technology. This article focuses on the need to cut a specific shape, ordered by the Department of Inorganic, Analytical Chemistry and Electrochemistry, Faculty of Chemistry, Silesian University of Technology, Gliwice, Poland, and the material provided by the department. The defined cutting shape is imposed due to the needs of the industry [28]. The cut shapes are to be used as elements for spacers, assembled in the appropriate order and arrangement, to be produced individually and in small batches, hence why the appropriate cutting of the ordered elements is so important. The supplied material on which the tests and final cuts were performed is a composite—polyvinyl chloride film with plasticizers. The power of the laser mounted to the robot is 20 W. While carrying out research related to laser cutting, certain problems were encountered, which we tried to eliminate step by step.

**Figure 4:**
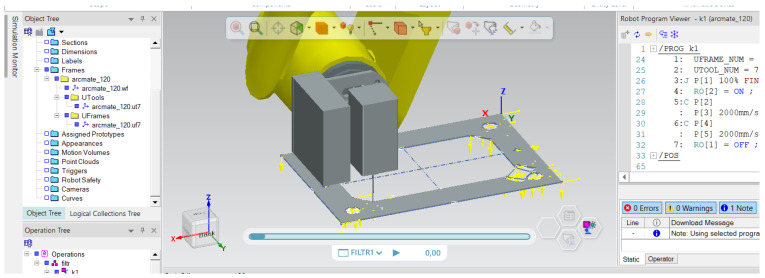
A view of the programmed cutting path in the CAM program ver. 2206.

The first stage of the laser cutting process was the appropriate programming of the cutting path, which was imposed and required by the client. A view of the programmed cutting path is shown in Figure 4. The working area is 8 by 5.5 cm.

This stage was first carried out in a program dedicated to offline programming, and then the program was tested on a real station with a robot. The ability to program the path increases the flexibility of making individual cuts because the path can be freely modified and programmed, according to the customer’s requirements. Additionally, the robotization of laser cutting itself allows you to obtain elements in which the material surface is important; i.e., it should be damaged as little as possible, so they can be used in various processes where mechanical or thermal strength is important, such as in chemical processes. At the same time, the cutting process itself is repeatable and identical with each attempt, which is also of great importance. An additional advantage of using laser cutting is that it accelerates the process of obtaining the cut element, but also the required shape and repeatability, as mentioned above, because the material was previously cut by hand.

## 5. Research Problem Regarding Cutting at the Station

The first major problem with actually cutting material based on a programmed cutting path was the constant distance of the laser from the material. Holding the laser manually was not taken into account because cutting accuracy, especially on rounded edges; repeatability; and flexibility in changing the cutting path are important here, hence the mounting of the laser on the robot arm. Thanks to this, a constant distance was achieved during the entire cutting process in the laser-serial-material system and a constant speed of movement of the laser arm. This also allows you to make repeated cuts of the indicated shapes, without the need to program the robot each time. This also makes it possible to return to the loaded program at a later time.

The above issue is also related to setting the coordinate system for the robot arm that carries out the cutting process on the technical table. Each time the table is moved, the coordinate system must be reset. The cutting process itself always took place at a focal length specified for a given material (its size influenced the possible formation of burns on the edges). The most frequently used focal length was 32.8 mm. The cutting speed (laser head movement) was used in the range of 5 to 20 mm/s. The burst of speed resulted in fewer edge burns.

**Figure 5:**
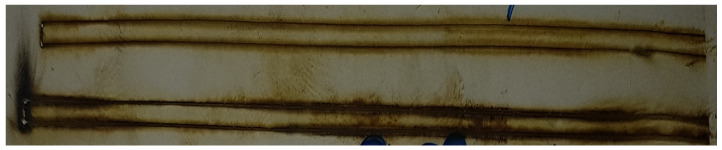
A view of the test cutting of the investigated polymer film.

Another major problem was determining the laser power. The 20 W laser itself cut the material, but also burned it. Hence, with the specially designed and manufactured laser regulator, the regulation increases the possibility of cutting various types of materials, i.e., in this case, a composite—polyvinyl chloride film with plasticizers. The task was more difficult because the material was transparent, which made it more reflective of the laser light. An example of one of the cutting tests carried out according to literature parameter settings is shown in Figure 5. In this photo, you can see a very large number of substrate oxidation elements on the edges, which makes it impossible to use this element in an ion filter. The level of gasket contamination was checked separately at the Chemistry Faculty as part of separate tests.

The test was performed on parameters described in the literature. Unfortunately, in this case, large burns are visible at the cutting site. Hence, the construction of a station would support the performance of this task by the imposed quality requirements and the analysis of the material to minimize the resulting burns in subsequent attempts.

**Figure 6:**
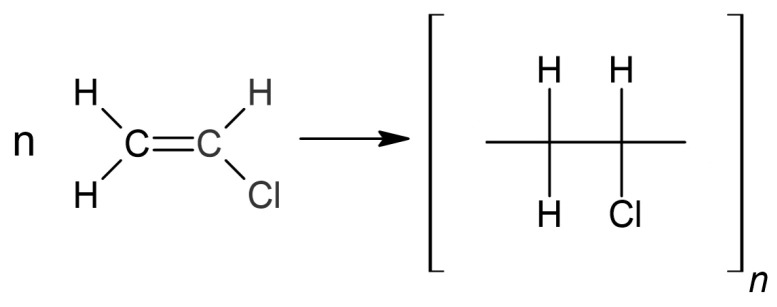
Polymerization of polyvinyl chloride.

The material on which the cutting tests were carried out is a polymer, more precisely a polyvinyl chloride film with plasticizers (PVC), which is used in many industries. Polyvinyl chloride is a durable and widely available raw material, produced in hard (unplasticized) or soft (plasticized) forms, in the form of foil, profiles, and pipes. It can be widely used due to several advantages, including that it does not emit an unpleasant odor, it is non-toxic, it works well at both high and low temperatures, and it is not susceptible to moisture, acids, and alkaline compounds [29]. The chain polymerization itself for this compound proceeds as shown in Figure 6.

For polymers, including the one used in the research, the so-called degradation reaction is observed, especially since heat is generated during cutting. However, during the degradation itself, two types of reactions can be distinguished: one-stage and chain. Due to laser cutting and thermal initiation, a chain reaction occurs with this material. The reaction is characterized by the fact that as the result of this reaction, the products are formed that are capable of spontaneously reacting with subsequent substrate molecules. However, when the phenomenon occurs continuously, the speed of the entire process is then multiplied [29].

**Figure 7:**
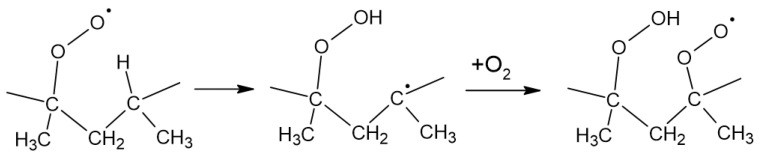
Propagation in polymers.

In polymers, propagation also occurs intramolecularly. This phenomenon can be described as in Figure 7.

Additionally, due to laser cutting and heat generation during the process, this material is subjected to degradation, as previously mentioned, but more precisely it is the so-called thermal degradation. This phenomenon occurs when the polymer changes its chemical properties under the influence of increased temperature. The process itself takes place without the use of chemical agents. However, it should be noted that polymeric materials are rarely chemically pure and usually contain additional ingredients such as dyes, fillers, stabilizers, or impurities. These additional ingredients contained in the material can react with the polymer at elevated temperatures.

Polymers are usually stable up to temperatures of around 2000 °C, but at temperatures of around 1000 °C, the molecules break down into small fragments, such as free radicals or free H_2_, CO, and CO_2_ ions. The chemical changes that occur during heating result in two phenomena: C-C chemical bonds in the main or side chain are broken. This is manifested by a decrease in molecular weight and the release of low-molecular-weight gas products. A characteristic product of polymer combustion is soot. The amount of soot produced during polystyrene combustion can be reduced by carrying out the process in a slightly oxidizing atmosphere and, depending on the amount of oxygen supplied, the soot is oxidized to CO and CO_2_.

In addition to adjusting the laser power, adjusting the travel speed of the robot arm with the laser also turned out to be helpful. This setting allowed for a slightly better cutting effect, which allowed for both cutting with less burnt edges and cutting the film itself.

The third problem was the use of an appropriate substrate for the cut material. On the one hand, the use of an appropriate board protected against cutting or burning the technical table, but also helped to safely carry out all tests. There was also a mesh on the plate, which made it easier for gasses to escape.

They introduced solutions to problems that arose during the construction of the site and the first tests allowed the site to be improved and tests to be carried out. These tests are dictated by the need to obtain appropriate elements with assumed shapes, cut out in foil, which are then used in special conditions, e.g., in the chemical industry. Due to the requirements imposed by the subsequent use of the elements, it was decided to use laser cutting, but also in this case, it was necessary to modify the parameters available in the literature and, based on the cutting effects and quality assessment, the settings and parameters were improved to obtain the desired effect. At the same time, an additional advantage used in these studies was the placement of the laser on the robot arm, thanks to which the shapes of the cutting lines can be flexibly changed and programmed, depending on the need.

## 6. Comparison of Cutting Effects at the Laboratory Station

**Figure 8:**
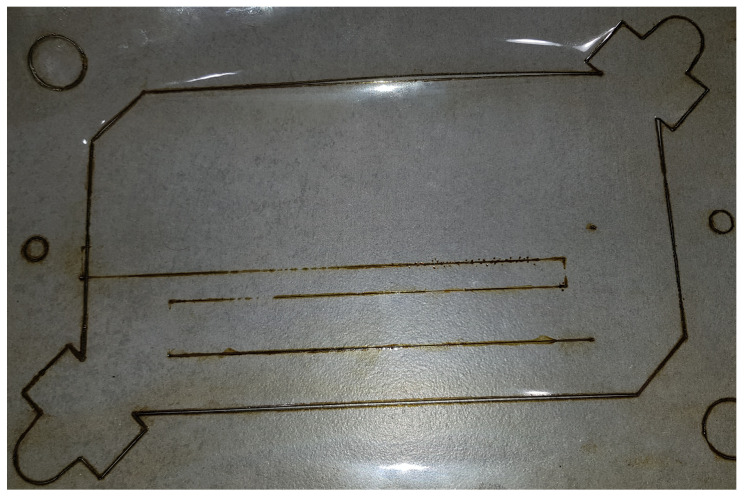
Research No. 1, the effect of film cutting.

**Figure 9:**
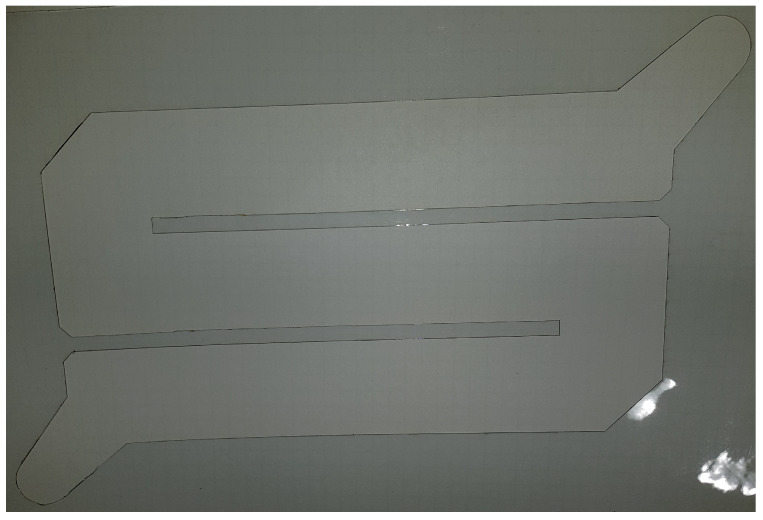
Research No. 2, the effect of film cutting.

The results of the tests—laser cutting of the programmed path—are described as test No. 1 and test No. 2 and are also shown in the two drawings below (Figure 8 and Figure 9).

### 6.1. Research No. 1

Laser power at 100% was used during this study. The cutting speed set on the robot is 5 mm/s, using automatic cutting. Air blowing at the laser beam was not used in this study (blowing causes a greater tendency for the laser to burn the edges). What is also important is that the cutting took place without the glass placed on the foil. The cut material was a transparent polyvinyl chloride film with plasticizers (Figure 8).

As a result, the intended goal was achieved in the form of a cut shape by the programmed cutting path. However, burnt material can be seen on the entire cut element. There are several possible reasons for this, e.g., too low cutting speed, too high laser power, or no air being used at the station.

The set parameters, which are included in the literature, pay attention to the laser setting, the distance of the laser beam from the cut material, and the type and color of the material. However, these parameters were not sufficient, taking into account the obtained effects, in particular that the constructed station had a laser power regulator, but also the speed of movement of the robot arm with the laser was modified, and air was connected. As you can see, the problem was not that the material was transparent, because we managed to cut the film over the entire surface, by the required shape.

To eliminate burns on cut edges, due to quality requirements, several solutions were used. This is important because overburns may affect the research for which the cut elements are to be used, namely filters, and hence the problem of eliminating such large overburns becomes so important.

Therefore, another study was carried out.

### 6.2. Research No. 2

Laser power at 100% was also used during this study. The cutting speed set on the robot is still 5 mm/s when automatic cutting is used. The higher speed meant that sometimes the film was slightly undercut. However, during this study, first of all, the connected system with air blowing near the laser beam was activated. Also, during this test, cutting was performed without the glass placed on the foil. The cut material, although also in a different shape, was a transparent polyvinyl chloride film with plasticizers (Figure 9).

As a result, the intended goal was achieved in the form of a cut shape by the programmed cutting path. Only in specific places of the cut element can you notice slight edge burns. Comparing the effects of cutting in both studies, study No. 2 was much better. The change in the form of additional air blowing at the laser beam meant that the cutting line was not burnt, as in test No. 1.

**Figure 10:**
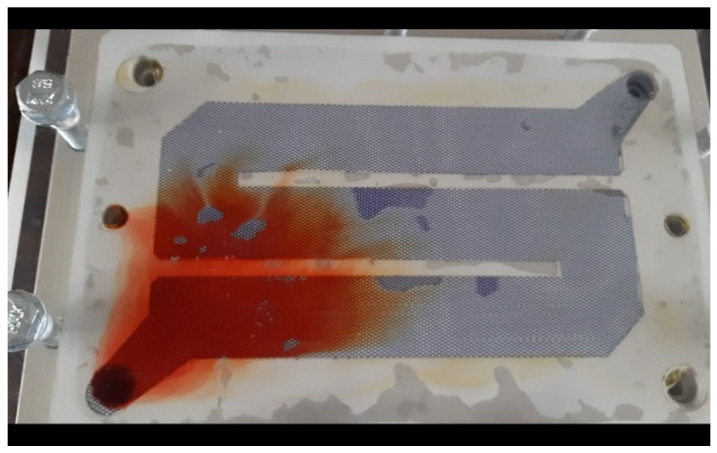
Using cut-out elements as filters.

Also, the below image shows the use of cut items as gasket spacers. An example is shown in Figure 10. It should be emphasized that separate tests at the Department of Inorganic, Analytical Chemistry and Electrochemistry, Silesian University of Technology, Gliwice, Poland confirmed that the cut elements (gasket spacers) met the chemical purity criterion. The amount of remaining substrate oxides did not affect the quality of operation of gasket spacers made on the basis of laser-cut polyvinyl chloride films.

The research has shown that the use of a laser as a method of cutting individual elements can give very interesting and satisfactory results. The need to cut elements for the chemical industry also resulted in the use of available individual solutions, which, after integration and programming, enable the execution of special orders, including customized production. The ability to perform tasks using a laser mounted on the robot arm allows you to cut various programmed cutting paths, making it possible to flexibly change the cutting line. This makes it possible to produce many elements both in individual orders and in small series. An additional advantage is the cost-effectiveness of the solution, which enables the use of available solutions from the department. However, during the research, the first focus was on the construction of an appropriate station and the integration of the robot arm–laser. In the next steps, a regulator was also built that is used to regulate the laser power. The results obtained are promising and allow for the continuation of the research.

It should be stated that experiment No. 1 represents an approach consistent with the descriptions contained in the literature. Based on the parameters indicated in the literature, the required dimensional accuracy of the cut element was achieved while maintaining the expected process efficiency (cutting speed). However, chemically, the cut polyvinyl chloride films turned out to be defective because they contained substrate oxides that negatively affected their performance in the ion filtration system. Experiment No. 2 represented an approach developed as part of the research, which differs from the indications in the literature on the subject due to the specificity of the cut-out elements. The cut element already met both the dimensional and chemical purity criteria. The aim was to develop a cutting technology that would not require the use of protective glass on the cut foil (isolation from the air supply to the cutting site). However, the glass significantly limited the power of the laser beam reaching the cutting site.

## 7. Future Works and Discussion

The research in the form of laser cutting and its effects gave promising results. These results are the basis for further research and the possibility of expanding the station, especially since the solution is flexible and can be used for small-batch and customized production, and the laser cutting used in a specific material meets quality requirements and can also be used in the chemical industry.

However, to carry out further research, further tests will be performed, which will concern, among others,

- Further research taking into account the diversity of cutting materials;
- Further research on changes in laser power regulation;
- Changes to the robot’s cutting speed settings;
- The application of microscopic examination of the obtained cutting effects;
- The expansion of the site;
- Possibility of cutting automatically, by the saved cutting trajectory program;
- A more detailed examination of the effect of cutting speed on the char formation;
- The examination of the structure of the material under a microscope and the occurrence of charring;
- Testing of materials for the production of coatings resistant to mechanical and thermal wear and corrosion;
- Further work related to the use of modern/innovative techniques for obtaining the required materials, resistant to mechanical and thermal wear and corrosion;
- Further research on the use of modern/innovative materials acquisition techniques and their economics.

The development of technology provides many opportunities to produce materials and obtain products in various ways. The problems that arise in these areas concern aspects related to the economics of the solutions used, the possibility of implementation, and the effects of the solutions obtained. Due to innovative technologies for the production of various types of products, in addition to welding, surfacing, and applying appropriate coatings, 3D printing and laser cutting are also very popular technology. However, the selection of technologies requires knowledge of where these methods, materials, and effects obtained can be used. Not all components can be used in all industries due to, among others, corrosion or melting of the material surface or its edges [30]. Laser cutting offers many possibilities for producing materials, especially if other technologies fail. The solution used in this article, which is an innovative combination because the laser is mounted on the robot arm, provides more manufacturing possibilities. Additionally, to increase production possibilities, especially in the era of customization, the use of a laser power regulator allows for an increase in the number of different materials to be cut. The laser cutting used is precise, does not tear the edges of the material so much, and allows for cutting various precise shapes in the material. Another advantage of mounting the laser on the robot arm is that it allows you to use the available infrastructure of small- and medium-sized enterprises where it is not possible to invest in additional machines. This is a more economical solution. Additionally, the written program can be used multiple times, on various materials, which also increases the company’s competitiveness. However, the problem is determining the laser power depending on the selected material and the cutting speed. These parameters should be appropriately selected for the material being cut to avoid charring of the edges or undercutting of the material. In particular, heat is generated during cutting, and some materials, i.e., the material described in this article, produce soot due to the heat release, which is why burns can be observed on the edges.

## 8. Conclusions

Research has shown that the use of a laser as a method of cutting individual elements can give very interesting and satisfactory results. The need to cut elements for the chemical industry also resulted in the use of available individual solutions, which, after integration and programming, enable the implementation of special orders, including non-standard production. As indicated earlier, the aim of this research was to obtain the required degree of purity of the gasket spacers (minimum amount of substrate oxides). Research conducted at the Faculty of Chemistry confirmed that this criterion was met. Secondly, the ability to perform tasks using a laser mounted on the robot arm allows you to cut various programmed cutting paths, making it possible to flexibly change the cutting process. Thanks to this, it is possible to produce many elements both on individual orders and in small series. An additional advantage is the cost-effectiveness of the solution, which allows for the use of solutions available at the department. However, during the research, the first focus was on the construction of an appropriate station and the integration of the robot arm–laser. In the next steps, a regulator was also built that is used to regulate the laser power. The results obtained are promising and allow for further research. When setting the parameters given in the literature, pay attention to the laser setting, the distance of the laser beam from the material being cut, and the type and color of the material.

However, these parameters were not sufficient, taking into account the results obtained—the constructed station had a laser power regulator, but the speed of movement of the robot arm with the laser was also modified and air was connected. As you can see, the problem was not that the material was transparent, because we managed to cut the foil over the entire surface to the required shape. To eliminate burns on cut edges, due to quality requirements, several solutions were used. Ultimately, the operating parameters were a cutting speed of 5 mm/s and laser focal length of 32.8 mm. This is important, as mentioned above, because burn-throughs may affect the tests for which the cut elements, i.e., filters, are to be used, and hence the problem of eliminating such large burn-throughs becomes so important. As a result, the intended goal was achieved in the form of a cut-out shape in accordance with the programmed cutting path. Only in some places of the cut element there are slight edge burns visible. Compared to the cutting effects in both studies, study 2 was significantly better. The change in the form of additional air blowing onto the laser beam meant that the cutting line was not burned out as in test No. 1.

## Data Availability

The raw data supporting the conclusions of this article will be made available by the authors on request.

## Abbreviations

CFRP	carbon fiber-reinforced polymer—a composite reinforced with carbon fiber and a polymer cover.
MDF	medium-density fiberboard—medium-density fiberboard made of wood fibers. It is the basic material for the production of furniture and interior finishing products such as decorative strips and wall panels.
PC	polycarbonates—a group of polymers from the polyester group, formally carbonic acid esters.
PMMA	polymethyl methacrylate—a polymer from which acrylic glass is made.
PP	polypropylene—an organic chemical compound, and a polymer from the polyolefin group, composed of units with the formula −[CH_2_CH(CH_3_)]−. It is obtained as a result of the low-pressure polymerization of propene.
PVC	polyvinyl chloride—a synthetic polymer from the group of vinyl polymers, obtained by the polymerization of vinyl chloride.
SME	small and medium enterprises.
